# Dense single nucleotide polymorphism testing revolutionizes scope and degree of certainty for source attribution in forensic investigations

**DOI:** 10.3325/cmj.2024.65.249

**Published:** 2024-06

**Authors:** Sammed N. Mandape, Bruce Budowle, Kristen Mittelman, David Mittelman

**Affiliations:** 1 Othram Inc., The Woodlands, TX, USA; 2 Department of Forensic Medicine, University of Helsinki, Helsinki, Finland; 3 Forensic Science Institute, Radford University, Radford, VA, USA

## Abstract

The field of forensic DNA analysis has experienced significant advancements over the years, such as the advent of DNA fingerprinting, the introduction of the polymerase chain reaction for increased sensitivity, the shift to a primary genetic marker system based on short tandem repeats, and implementation of national DNA databases. Now, the forensics field is poised for another revolution with the advent of dense single nucleotide polymorphisms (SNPs) testing. SNP testing holds the potential to significantly enhance source attribution in forensic cases, particularly those involving low-quantity or low-quality samples. When coupled with genetic genealogy and kinship analysis, it can resolve countless active cases as well as cold cases and cases of unidentified human remains, which are hindered by the limitations of existing forensic capabilities that fail to generate viable investigative leads with DNA. The field of forensic genetic genealogy combined with genome-wide sequencing can associate relatives as distant as the seventh-degree and beyond. By leveraging volunteer-populated databases to locate near and distant relatives, genetic genealogy can effectively narrow the candidates linked to crime scene evidence or aid in determining the identity of human remains. With decreasing DNA sequencing costs and improving sensitivity of detection, forensic genetic genealogy is expanding its capabilities to generate investigative leads from a wide range of biological evidence.

Human identification using various DNA typing methods has become routine in several forensic applications, such as parentage testing, identifying missing persons in individual cases or mass disasters, and determining the source of biological evidence from a wide range of criminal cases (1-6). Alec Jeffreys pioneered DNA typing in 1985 by assaying repeat-sequence markers, known as variable number of tandem repeats (VNTRs), across the human genome, which, when combined, were reported to be individual-specific (7). Before this breakthrough, forensic cases relied on serological-based markers, which were relatively unstable, not present in all tissues, and had poor discrimination power (8). In contrast, DNA is a more stable molecule and comprises markers or loci that are sufficiently polymorphic to enable much higher resolution and power of discrimination.

In VNTR-based forensic testing, specific VNTR loci, known as minisatellites (9,10), were assayed with restriction fragment length polymorphism (RFLP) (11-13) or with the polymerase chain reaction (PCR)-based methods. VNTR analysis was ground-breaking in the early days of forensic DNA testing and was crucial in several high-profile cases, ushering in the discipline of forensic genetics. However, RFLP methods required relatively larger quantities of input DNA, in the range of 20-100 ng (13). Additionally, VNTR analysis was limited because the repeat units were relatively long. Thus, template DNA had to be relatively intact.

Although VNTR target enrichment by PCR afforded a substantial gain in sensitivity of detection (to the sub-nanogram range) (14,15), the use of these markers was relatively short-lived. The long-repeat targets were susceptible to the vagaries of PCR amplification, which resulted in substantial differential amplification of alleles and notable rates of allele drop-out. Soon after the implementation of VNTR/PCR, a better suited approach, which gained overwhelming acceptance, was to amplify short tandem repeat (STR) markers by PCR. STRs (16,17) contain shorter repeat units than do VNTRs, which makes them more robust to enrichment by PCR. The implementation of STR/PCR methodologies revolutionized forensic DNA analysis (18-20), leading to higher success rates in obtaining DNA profiles from forensic samples, especially those that are of low quantity or low quality. As a result, STRs replaced VNTRs as the primary markers in forensic DNA testing, and became the cornerstone of the Combined DNA Index System (CODIS) (21). CODIS has been an invaluable tool for developing leads for potential suspects; and even when a source lead cannot be developed because the donor is not in the database, CODIS can link crime scenes by using common DNA profiles derived from evidence.

The field of forensic genetics, again, is on the cusp of a revolution as it shifts from the analysis of small numbers of markers to the analysis of large numbers of markers, ie, single nucleotide polymorphisms (SNPs), which span the entire genome. SNPs, variants at single genomic positions, have gained substantial prominence over the last two decades as an important source of genetic markers that may be better suited for the analysis of challenged samples. Although there is increased recent interest, SNPs are not novel markers for analysis of forensic samples. In the late 1980s through the mid-1990s, SNPs were the primary markers assayed by PCR-based methods. Those methods were HLA-DQA1 (22,23) and Polymarker (24) detected by allele-specific oligonucleotide probe hybridization, as well as Sanger sequencing used for the analysis of the hypervariable regions of the mitochondrial genome (25-27). Unlike the current construct of STR kits, which are limited in panel size due to the throughput of capillary electrophoresis (CE)-based methods, SNP panels with today’s advanced technologies can include hundreds of thousands to millions of markers, providing much higher resolution and specificity in genetic profiling. The higher resolution and sensitivity allow for greater discrimination power in associating individuals who are not closely related as well as for direct comparisons. Additionally, advancements in genotyping and sequencing technology have made SNP testing more cost-effective. These attributes make SNP-based methods increasingly attractive for forensic investigations, particularly when STR analyses and government-owned national DNA databases fail to generate leads. This article describes SNP markers, their characteristics, and the technological advancements that enable substantially high-throughput analysis facilitating alternate methods for source attribution.

## The evolution of forensic DNA profiles

### STR-based DNA profiles

Since the 1990s, STRs have become the primary currency of the forensic genetics community (18-20). STRs are characterized by a high mutation rate, estimated to be approximately 1000 times higher than other genomic sites, which in turn contributes to their diversity (28). This inherent genetic variability results in high heterozygosity and the presence of multiple alleles at STR loci, contributing to their high power of discrimination for individual identification capabilities in forensic applications. The number of STR loci employed directly correlates with discrimination power. Moreover, the smaller size of STR loci than of their predecessors, the minisatellites, made them amenable for efficient amplification by PCR, which exploited the exquisite sensitivity of detection.

Forensically-relevant STRs exhibit a diverse range of allele frequencies in all populations studied, and this variation has been extensively documented in the literature and in databases such as STRBase, popSTR and STRider (29-31). These resources are a wealth of population data for estimating the rarity of a DNA profile within and among specific populations, thereby providing law enforcement and the judicial system with critical insight into the weight of DNA evidence. An inclusion across all interpretable loci from a comparison between evidence and a person of interest necessitates a statistical calculation. One statistical approach is the random-match probability - the likelihood that a randomly chosen individual from the relevant population(s) would share this profile. This probability helps evaluate whether the DNA evidence is common or rare, and the rarer it is infers a remote chance of coincidence. The lower that probability, the stronger the implication that the samples share a common source. An alternative statistical approach is to calculate the likelihood ratio (LR), which compares the probability of the DNA evidence under two mutually exclusive competing hypotheses. For example, a LR could be the probability of the genetic data if a human remains sample belongs to a specific pedigree as opposed to the remains being unrelated to the specific pedigree. The greater the odds, the greater the support for a particular hypothesis.

Early on, STRs were characterized by size-based separation using slab gel electrophoresis techniques (14). However, this method was cumbersome and consumed a lot of time and resources. A substantial improvement to STR analysis was higher-throughput CE (32,33). CE allowed multiplexing of STR loci (34,35), reduced sample consumption, and increased resolution in a semi-automated fashion. Massively parallel sequencing (MPS) provides much greater throughput than the CE such that thousands to millions of loci can be analyzed simultaneously (36-40). This capability increases the detectable diversity of the STRs within repeat motifs as well as in their flanking regions (41-43). The comprehensive sequence variation detected by MPS improves the power of discrimination, especially with partial STR profiles, enhances mixture deconvolution, and can assist in kinship identification but still only as far as first-degree (parent-child [PC] or full-sibling [FS]) relatives. Consequently, commercially available MPS forensic STR kits have been validated and are beginning to be implemented by forensic laboratories (44-47).

### Limitations of STR-based DNA profiles

While STR-based methods have been quite successful and will remain essential for forensic DNA testing for the foreseeable future, their reliance on developing investigative leads within government-maintained national forensic DNA databases also has significant limitations. Numerous forensic cases remain unsolved due to the reliance on database searches with STR profiles, which leaves investigations at a standstill. If the donor of crime scene evidence is not in the database, then the current database search system cannot generate a hit and there is no investigative lead. Additionally, this issue is particularly pronounced in cases involving unidentified human remains (UHRs), where the antemortem reference samples and family reference samples may be scant. Additionally, forensic STR profiling is not useful for identifying unknown suspects whose profiles are not yet included in the databases. When database searches yield no leads, cases can turn cold quickly, which allows perpetrators to evade justice and potentially continue their criminal activities.

STR loci also come with technical constraints that are more pronounced with forensic DNA, which tends to be degraded, chemically damaged, or present in extremely low quantities (48-51). In cases of degraded DNA, the amplification of STR loci may be challenging, leading to incomplete or no profiles. Additionally, forensic samples often contain mixtures of DNA from multiple individuals, further complicating the interpretation of STR profiles. Stutter artifacts generated during PCR can present challenges during mixture interpretation (52). Additionally, the high mutation rate for forensically relevant STRs can confound first-degree (PC/FS) kinship analysis (53). Other methods have been explored to exploit STRs, such as familial searching and Y-chromosome STRs/surname associations (54-57). However, familial searching using STRs generally extends to PC and FS relationships. Moreover, very few countries have included Y STRs in their national DNA databases and thus cannot make use of lineage associations to help identify the source of crime scene evidence via a database search.

### SNP-based DNA profiles

The molecular characteristics of SNPs render them particularly advantageous for forensic applications compared with STRs. SNPs exhibit a significantly lower mutation rate, estimated at approximately 1 in 100 million per replication (58), in contrast to STRs, which have a mutation rate of about 1 in 1000 (28). This lower mutation rate of SNPs reduces complications often encountered with STR analysis in kinship cases. Moreover, the analysis of distant familial relationships becomes uninformative when parental DNA samples are absent (59), which is a less pronounced limitation with SNP-based analyses due to their greater stability over generations compared with STRs.

One of the foremost benefits of SNPs is their presence in smaller DNA fragments compared with STRs, making them particularly advantageous for analyzing highly degraded DNA samples, which are common in forensic evidence. SNPs can be detected in fragments shorter than 100 bp, which enables genotyping from samples where STR analysis may fail or yield incomplete profiles. For example, Kieser et al (60) describe a SNP panel comprised of ~ 50 bp amplicons. Furthermore, SNP analysis is not affected by stutter artifacts, a common issue with STR alleles, thereby simplifying, in part, the analysis and interpretation process associated with mixtures.

While SNPs are less informative per marker compared with STRs, their widespread occurrence across the human genome - approximately 1 in every 1000 base pairs - provides substantial genetic variation. These vast numbers of SNPs can be analyzed simultaneously using high-throughput genotyping or sequencing. This technological leap, which gained momentum in the 2000s, has revolutionized forensic casework, where degraded, low-quantity template DNA is often encountered.

SNPs should not be lumped into a general category as a single marker type. It is better to classify SNPs based on their practical forensic applications, such as biogeographical ancestry analysis, determination of physical traits, identity testing, and kinship analysis (61-64). SNPs that determine the ancestry of an individual, originally termed ancestry informative markers (AIMs), differ notably in allele frequencies between population groups (61-63) and initially were proposed to indirectly infer phenotypic traits of a person of interest. Generally, population affinity, based on major population groups, could indicate in a limited fashion skin pigmentation, hair color, and eye color. Since the initial introduction of AIMs, panels have been developed that can resolve population affinity in greater resolution (65-67). Subsets of these biogeographical SNPs have been included in current MPS-based kits (68-71) and are being used to estimate population affinity in some forensic laboratories.

Instead of using surrogate AIMs to infer phenotype at a gross level, a direct method with phenotypic SNPs allows more precise trait prediction. Identification of phenotypic SNPs began more than 20 years ago focusing on eye color and skin pigmentation (72-75). The first forensic foray into estimating a visible phenotypic trait, red hair color, was developed by Grimes et al (76), who screened 12 melanocortin 1 receptor variants to provide investigative leads about the possible appearance of the donor of an evidence sample. However, the first major impact of phenotypic SNPs in the forensic arena was the development of a six-SNP panel by Walsh et al (77), called the IrisPlex, to predict blue and brown eye color. Since then, the capability of phenotypic SNPs to identify markers directly related to traits has progressed in their utility to predict visible traits, such as facial shape, skin pigmentation, eye color, hair color, baldness, and freckles, to name a few (78-81). This approach has formally been defined as a subdiscipline called forensic DNA phenotyping (82).

Early SNP typing techniques, such as the SNPforID 52-plex assay for identification and the 32-plex assay for ancestry determination, were designed to integrate with CE instrumentation already in use (for STR testing) within forensic laboratories (83,84). However, these CE-based approaches did not generate quantitative data, which limited their utility with mixed samples. They were also labor-intensive and had limited throughput, all of which hindered the full potential of SNPs for human identification.

Over the past two decades, significant technological advancements have mitigated many of these limitations. Microarray-based SNP genotyping and MPS, along with computational tools for large data set analysis, substantial cost reductions in sequencing, and the rise of genetic genealogy through direct-to-consumer companies and hobbyists have collectively enabled the exploitation of SNPs for human identification.

One argument raised against embracing SNPs is that, on a per-marker basis, they are inherently less informative than STRs, a fact attributed to the biallelic nature of most SNPs. This limitation has already been overcome as there are vast numbers of SNPs across the human genome (about 1 in every 1000 base pairs), and they can be simultaneously analyzed cost-effectively by MPS. Only around 50 independent SNPs, each with minor allele frequencies between 0.20 to 0.50, are necessary to match the discrimination power of 10-15 multi-allelic STRs typically used in analyses (85). Yet with MPS, hundreds of thousands to millions of SNPs can be analyzed in a single assay. Thus, the power of discrimination is orders of magnitude higher than that of any STR kit on the market today.

### SNP-based DNA profiles and forensic genetic genealogy

Forensic genetic genealogy (FGG) has emerged as a powerful tool in forensic investigations, particularly in cases involving UHRs or unsolved criminal cases. By combining SNP profiles with genealogical data from public databases or commercial genetic genealogy services, investigators can establish familial relationships and generate investigative leads in cases where traditional methods have been unsuccessful.

**Figure 1:**
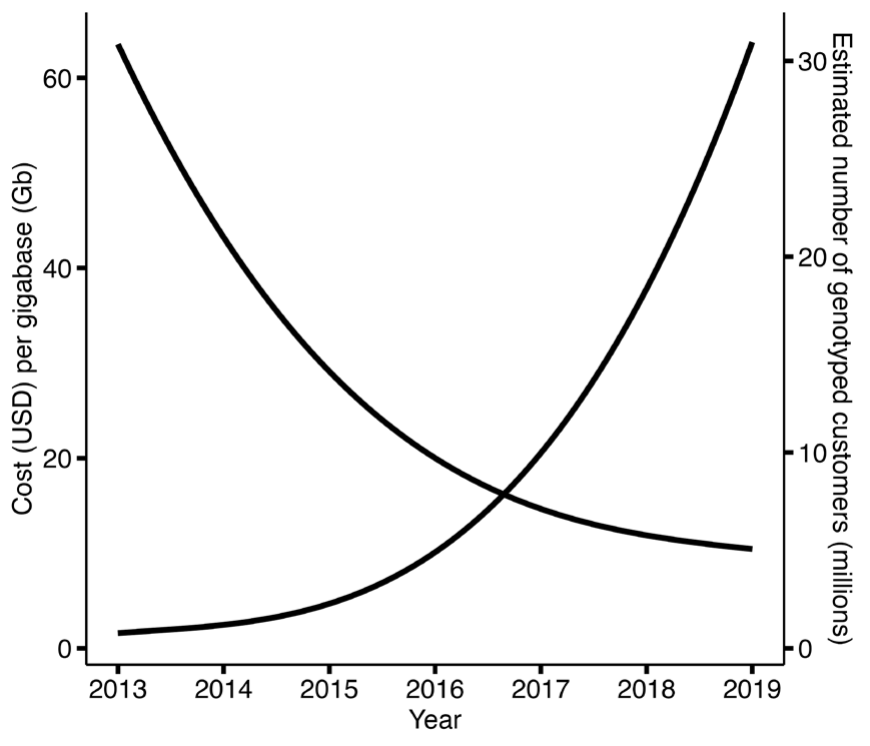
The reduction in cost (in US dollars) per gigabase for DNA sequencing and the concomitant increase in the number of individuals who were genotyped from 2013 to 2019.

The emergence of FGG in 2017 and 2018 was not incidental (Figure 1). In the last decade, two key developments have played a significant role. First, the cost of sequencing has decreased dramatically, which has made it more accessible for routine use in forensics and other fields (86). Second, there has been rapid growth in the number of people who have undergone consumer DNA testing (87,88). For example, in 2013 less than a million people worldwide were genotyped using consumer DNA tests. By 2019, the same figure was more than 30 million. While FGG investigations are limited to consumers who consent to use of their SNP profiles for developing investigative leads, this fraction of the total consumer profiles available is growing as well.

An early example of success with FGG is the identification of the Golden State Killer in 2018, where investigators used a SNP profile from crime scene DNA to identify distant relatives of the donor of the biological evidence through genetic genealogy databases (89). By building family trees and tracing common ancestors, investigators were able to narrow down their search to a suspect, Joseph James DeAngelo, who was subsequently arrested and charged with multiple crimes.

Another notable example is the Carla Walker murder case. Carla Walker was a 17-year-old girl who was abducted in February 1974 in Fort Worth, Texas. Her body was found three days later in a culvert. The case remained unsolved for decades until a breakthrough in which FGG led to the identification and arrest of a suspect, Glen Samuel McCurley, in September 2020. McCurley was charged with capital murder in connection with Carla Walker's death and convicted in 2021.

The Carla Walker case was significant for its outcome and for its legal implications. It was one of the first cases where FGG was presented in a courtroom setting. The use of FGG was subjected to a Daubert hearing, which is a legal process in the United States to determine the admissibility of scientific evidence, where several criteria are used by the judge, the gate keeper, to assess whether the methodology is sound and reliable. After being admitted by the judge, the trial proceeded, and FGG was presented by the scientific experts during the jury trial, where they were subjected to cross-examination. The case was appealed by McCurley, and an appellate court upheld the conviction. The successful use of FGG in the Carla Walker case set a precedent for this technology, highlighting its potential as a powerful tool in solving cold cases and bringing perpetrators to justice.

SNP profiles have also been instrumental in identifying UHRs. The National Missing and Unidentified Persons System (NamUs; https://namus.nij.ojp.gov) is now making use of FGG in UHR casework. NamUs is a national centralized repository and resource center for missing persons and unidentified decedent records. It also provides technology, forensic services, and investigative support to resolve missing persons and unidentified decedent cases. FGG has been instrumental in solving many NamUs cases, leading to the identification of previously unknown individuals.

In a recent and ongoing study (90), Dowdeswell found that as of December 2023, FGG has been publicly attributed to 318 criminal cases and 461 UHR cases. These numbers are an underestimate of the total number of forensic cases that have benefited from FGG. Many cases are not announced immediately, particularly if they are yet to be adjudicated, and some cases are resolved without public disclosure. Thus, the true impact of FGG in forensic investigations is likely far greater than what has been reported.

### Technologies for building DNA profiles for FGG

The emergence of FGG has revolutionized the field of forensic DNA analysis. The principal objective of FGG testing is to construct a detailed SNP profile that can be used for genetic genealogy and that allows for the identification of distant relatives. To achieve this power of kinship analysis, a sufficient number of SNP markers is required so that the profile is robust enough for inclusion in a genetic genealogy database and to enable the detection of genetic relatives. Close and distant genetic relatives are important for genetic genealogy. Distant associations are crucial for validating family trees, excluding irrelevant branches, and assisting in the selection of reference testing candidates. It is the combination of close and distant associations that allows for efficient construction and triangulation of family trees, ultimately aiding in the determination of an individual's identity.

Prior to genetic genealogy being used in forensics, genetic genealogy in the consumer DNA market predominantly relied on microarray-based genotyping. Microarrays are valued for their speed, simplicity, and relatively low cost. However, microarrays are less effective for forensic applications, as they require relatively large quantities of high-quality DNA to produce data that can be used for effective genealogical searches. For example, Illumina recommends 200 ng of input DNA for their Infinium Global Screening Array-24 v3.0 BeadChip, per their Infinium® HD Assay Ultra protocol. Microarray-based genotyping is also sensitive to DNA quality, and performance is reduced with degraded DNA inputs, which leads to diminished success in kinship inference (91,92).

Acknowledging these limitations, the FGG community has largely shifted toward MPS, which not only offers greater sensitivity, but also the capacity to analyze more complex and degraded samples (93,94). MPS can operate with substantially lower DNA quantities, depending on the assay and the library preparation kit protocol used, as different kits are optimized for specific sample types and sequencing applications.

While microarrays use a probe-based hybridization technique on a chip, MPS determines the entire sequence of millions to billions of DNA fragments simultaneously and rapidly. In the last decade, the dominant MPS method has been Illumina sequencing, which uses a method called sequencing by synthesis. In this technique, DNA fragments are attached to a flow cell surface and amplified to create clusters that contain identical sequences. Fluorescently labeled nucleotides are then sequentially added to the growing DNA strands, with each nucleotide type carrying a distinct fluorescent marker. The incorporation of these nucleotides is detected through imaging, which allows the sequence of the DNA fragments to be determined based on the observed color pattern. Notably, the Illumina chemistry can sequence read lengths shorter than 50 bp, which makes the system robust to DNA degradation and facilitates detection of SNP markers.

Early efforts to measure additional information with SNPs in forensic samples focused on using desktop sequencers, typically assaying up to 100 or so SNPs or by increasing the diversity of STRs to a degree (41,95). Larger targeted panels, ranging from 5000 up to 95 000 SNPs, have been developed to measure kinship up to fourth-degree relationships (for example, first cousin once removed) and sometimes fifth-degree associations (similar to that of a second cousin) (96-98). Whole-genome shotgun (WGS) sequencing stands out by enabling the analysis of over a million SNPs per good-quality sample (99), thereby extending the potential for kinship associations to the seventh degree (similar to that of a third cousin) and beyond.

Simulation studies have explored the relationship between the size of SNP marker panels and the sensitivity and recall rate for distant genetic relationships (100). In one recent study based on simulations but also experimental results, a target panel of 10 000 SNPs enabled kinship inference to fourth-degree relationships, with a sensitivity to fourth-degree relationships of about 95% (96).

**Figure 2:**
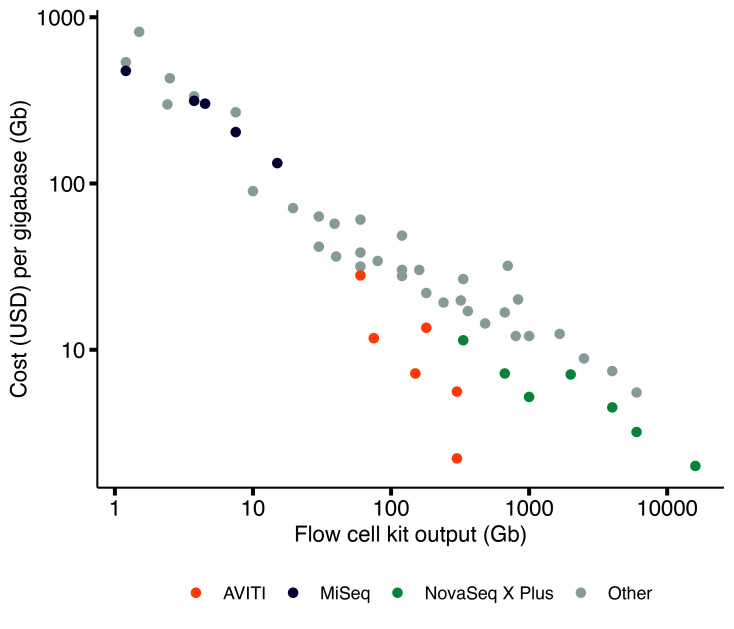
The tradeoffs in gigabase throughput and costs (in US dollars) of selected DNA sequencing platforms.

In forensic applications, the choice between targeted sequencing and WGS sequencing has traditionally been influenced by cost considerations and the specific needs of the investigation. Targeted sequencing focuses on specific SNPs, providing data for these genomic areas at an apparently lower cost. This approach is useful for analyzing specific genetic markers relevant to forensic cases, such as those used in DNA profiling or identifying familial relationships. However, as sequencing technologies advance and costs decrease, the gap between the expense of targeted sequencing and WGS sequencing is narrowing (Figure 2). Figure 2 illustrates that platforms and respective chemistries continue to improve in throughput with concomitant reductions in cost. In a cost-neutral scenario, WGS sequencing becomes increasingly attractive for forensic applications because it offers a more comprehensive view of the genome and supports kinship associations, especially more distant ones. Not only does WGS provide a larger number of SNPs, but it also reveals other types of genetic variations, such as insertions, deletions, and structural variants, which can be valuable in forensic investigations. These additional variations can offer more information for identifying individuals and understanding complex relationships. Other benefits include the potential for mixture deconvolution that likely will meet that of STRs. Currently, the majority of laboratories apply STR mixture deconvolution by probabilistic genotyping up to four-person mixtures (101). Since WGS comprehensively scans the genome and is quantitative, one can make use of a reasonably large number of tri-allelic SNPs and microhaplotypes to achieve similar mixture deconvolutions as with STRs (102-104). These markers exhibit higher discrimination power than a high heterozygosity biallelic SNP (ie, >0.50). Moreover, there are other data in WGS analyses beyond SNPs that have yet to be exploited, such as structural variants and the microbiome that could assist in investigations. For example, Toppinen et al (105) have shown that persistent viruses in femoral bone may provide information on the geographic origin of human remains.

## Concluding remarks

The forensic DNA field is at another critical junction to enhance analysis of a wide range of biological evidence, ie, embracing the revolution of SNPs, targeted sequencing, and preferably WGS sequencing, and the use of alternate databases. These technical advancements generate substantial whole genomic data to permit kinship associations as distant as the seventh degree and beyond. FGG makes use of these associations to facilitate genetic genealogy to effectively narrow down the candidates who can be the source of crime scene evidence or assist in the determination of the identity of UHRs. The large number of (cold) cases and UHRs lying dormant, as well as active cases, now have an opportunity to be resolved rapidly. While STRs and national databases will continue to be important tools for developing investigative leads, investigators no longer need to rely solely on developing DNA-based leads where the reference sample must be in the database. Notable successes, such as the identification of the Golden State Killer and the resolution of the Carla Walker case, highlight the impact of these advances on public safety and justice. FGG’s success could increase if the genetic genealogy databases expand and become more representative of the populations of various regions around the world that make use of this powerful tool.

As with any genetic technology, especially those employed in FGG, the benefits and limitations must be weighed, and proper governance is required. There is no doubt that FGG is a powerful tool for providing critical leads and helping solve previously intractable cases. It is essential to consider individual privacy to ensure the adoption of this invaluable tool (106-109). In most cases, with comprehensive genome scanning, genetic data can be associated with at least some clinical and subclinical traits. The best way to address privacy concerns with genomic data are through proper governance, security, accountability, and integrity (110).

The benefits of public safety and security, bringing resolution to victims, families, and communities, and developing leads in a cost-effective and rapid manner likely will drive the adoption of FGG. The overall value highlights the importance of careful investment and governance in leveraging these technologies for societal benefit.
